# An Integrated Predictive Impact–Enhanced Process Mining Framework for Strategic Oncology Workflow Optimization: Case Study in Iran

**DOI:** 10.3390/bioengineering12121288

**Published:** 2025-11-24

**Authors:** Mohammad Salehi, Raouf Khayami, Reza Akbari, Mirpouya Mirmozaffari

**Affiliations:** 1 Department of Computer Engineering and IT, Shiraz University of Technology, Shiraz 13876-71557, Iran; mo.salehi@sutech.ac.ir (M.S.); khayami@sutech.ac.ir (R.K.); akbari@sutech.ac.ir (R.A.); 2 Department of Industrial Engineering, Dalhousie University, 5269 Morris Street, Halifax, NS B3H 4R2, Canada

**Keywords:** process mining, oncology, predictive modeling, PM^2^, chemotherapy workflow, conformance checking, bioengineering, workflow optimization, predictive impact model (PIM)

## Abstract

Process Mining (PM) effectively diagnoses inefficiencies in complex healthcare workflows, such as chemotherapy protocols. However, current methodologies often remain retrospective or rely on loosely coupled simulations, leaving a critical methodological void: the inability to quantify the aggregate, system-wide operational impact of eliminating specific, diagnosed workflow deviations. This gap prevents decision-makers from forming evidence-based strategies for resource allocation. We address this by introducing the PM^2^–Predictive Impact Model (PIM) framework, a novel, fully embedded process-native methodology that unifies conformance checking, predictive monitoring, and quantitative scenario analysis within a singular, closed-loop structure. Using event logs from an Iranian Radiotherapy and Oncology Center, we modeled a normative seven-step pathway (Fitness = 0.97, Precision = 1.00) and identified high-impact deviations, including skipped approvals and resequencing, enabling a direct causal linkage between deviation categories and system performance. PIM simulation demonstrated that removing these deviations yields statistically significant reductions in managerially relevant KPIs: Cycle Time (8.00%) and Workload (6.00%), which were robust to parameter uncertainty (*p* < 0.001). The PM^2^–PIM framework thus transforms retrospective diagnosis into proactive, quantitatively justified strategic planning, providing oncology services with a reproducible, low-cost, and evidence-rich basis for prioritizing interventions and achieving sustained performance gains.

## 1. Introduction

Cancer treatment, particularly in oncology workflows, is both clinically demanding and operationally complex [1]. The administration of chemotherapy, a cornerstone of many cancer treatment regimens, involves an intricate series of steps that must be performed with precision and in a timely manner [2]. These steps typically include physician consultation, prescription generation, pharmacy preparation, nursing administration, and post-treatment monitoring [3]. Any deviation from the optimal sequence or timeline of these activities can lead to a cascade of negative consequences, including prolonged patient waiting times, inefficient allocation of valuable medical resources (such as physician and nursing staff, as well as specialized equipment), increased operational costs, and, most critically, potential compromises to patient safety and treatment efficacy due to delays or errors [4,5].

To address this inherent complexity and variability, Process Mining (PM) has emerged as a transformative analytical paradigm [6]. PM fundamentally bridges the established, structured principles of Business Process Management (BPM) with the data-driven, predictive capabilities of modern advanced analytics [7]. By systematically leveraging the comprehensive event logs automatically generated by existing healthcare information systems (e.g., Electronic Health Records (EHRs), Pharmacy Information Systems (PIS)), PM offers the unique capability to discover the actual, as-is workflows being executed on the ground, objectively assess their adherence (or conformance) against intended, normative process models, and subsequently identify specific, actionable opportunities for process improvement [8,9]. While Process Mining has demonstrably achieved significant success in optimizing processes within industrial manufacturing and various service sectors [10], its focused application within the highly specialized field of oncology particularly to quantify the performance impact attributable to specific workflow deviations remains relatively limited [11].

The application of Process Mining (PM) in healthcare has evolved significantly, transitioning from descriptive mapping to sophisticated diagnostic, and more recently, predictive, analytics. This evolution can be understood in distinct phases, as highlighted by a comparative review of the literature, which reveals a clear trajectory toward proactive decision support (Table 1).

**Phase 1: Process Discovery and Conformance Checking**

Early healthcare process-mining research concentrated on describing what truly happens within clinical workflows rather than assuming adherence to designed guidelines. Foundational contributions such as Rebuge & Ferreira (2012) established PM’s diagnostic power by reconstructing emergency-care pathways from event logs and exposing discrepancies between intended and observed behavior [12]. Fernández Llatas et al. (2015) advanced precision by integrating indoor-location data for surgical process tracking [13]. This descriptive stream soon extended to oncology: Rojas et al. (2016) and Savino et al. (2023) mapped real cancer-care sequences and measured model fitness yet remained confined to structural visualization of variances [11,14]. Recent tertiary reviews Santos et al. (2025) and Aversano et al. (2025) confirmed that discovery and conformance checking still dominate healthcare PM, particularly in oncology where noisy event logs hinder predictive linkage [15,16]. Mansur et al. (2024) represent the maturity edge of this phase: their comparative mapping of breast cancer treatment trajectories (chemotherapy vs. radiotherapy vs. combined) exemplifies PM’s descriptive depth but reveals an inability to quantify how much deviations affect outcomes [17]. This insight defines the first methodological bottleneck the field has mastered diagnosis yet lacks the quantitative instruments to measure severity or consequence. That limitation naturally motivates the next phase, which strives to transform qualitative deviation knowledge into measurable performance evidence.

**Phase 2: Retrospective Performance Analysis**

Building upon descriptive foundations, the second phase sought to answer, “Where and why do inefficiencies occur, and how strongly do they affect performance?”

Kurniati et al. (2018) pioneered this transition by mining the MIMIC-III dataset to quantify temporal bottlenecks along oncology-related pathways among the first explicit attempts to convert deviation frequency into data-driven delay metrics. Yet their analysis, while quantitative, remained retrospective: it measured variation without predicting improvement potential [18]. In Iran’s healthcare context, Atighehchian et al. (2022) deepened this approach by defining delay-measurement KPIs at national scale, translating PM outputs into hospital performance indicators (e.g., waiting-time deltas). This shifted PM toward operational benchmarking but still offered no built-in forecasting of how proposed corrections would change those KPIs [19]. From a methodological perspective, De Roock & Martin (2022, JBI) clarified the limitation by classifying healthcare PM applications into support, control, and configuration roles explicitly noting the absence of process-native systems for predictive or prescriptive analysis [20]. Organizationally, Rosa & Massaro (2024, Engineering) incorporated ML decision engines into a Process Mining Organization (PMO) framework for preventive medicine, extending PM toward AI-driven enterprise analytics. However, their architecture remained detached from the behavioral process layer, improving prediction of what decisions should be made but not how specific process deviations undermine efficiency [21]. In parallel, Samara & Harry (2025, Healthcare) hybridized PM with Kaizen principles, creating an iterative human–digital improvement loop. While effective for continuous refinement, its impact quantification stayed qualitative: capable of detecting and correcting deviations but unable to forecast aggregate benefits before implementation [22]. Taken collectively, the Phase 2 literature established a solid base for quantifying historical inefficiencies and embedding continuous-improvement mechanics. Yet all studies converge on one unresolved challenge: absence of a unified, process-native method translating deviation diagnosis into predictive, evidence-based forecasts of system-level gains.

This analytical ceiling marks the conceptual gateway to forward-looking analytics, setting the theoretical stage for the Predictive Impact Model (PIM), a framework designed precisely to operationalize that bridge between retrospective KPI analysis and quantitative forecasting.

**Phase 3: Toward Forward-Looking Analytics**

The third phase signifies the field’s evolution from retrospective explanation toward prospective simulation and prediction. Here, PM research bifurcates into two synergistic trajectories: (3a) Predictive Monitoring of Ongoing Processes, and (3b) “What-If” Scenario Simulation.

**Path 3a: Predictive Monitoring of Ongoing Processes**

This line of inquiry asks: “What will happen next in an active process instance?”

Teinemaa et al. (2018) laid the foundations by learning remaining time and outcome probabilities using supervised ML, proving feasibility but addressing only micro-level control [23]. Kratsch et al. (2021) broadened the paradigm through deep-learning comparison (LSTM, feed-forward NN vs. RF/SVM), achieving accuracy gains yet suffering from black box opacity the models predicted but could not explain which process factors drove risk [24]. Madau et al. (2025) implemented early-warning systems for emergency events, providing actionable real-time alerts but not evaluating systemic efficiency [25]. To connect prediction with understanding, Winter et al. (2024, npj Digital Medicine) hybridized PM and ML over decade-long mHealth datasets, showing that ML detects statistical patterns while PM contextualizes them through temporal-state transitions. This integration grounded AI predictions in interpretable process semantics [26]. Building upon this, Delgado et al. (2025, Discover Analytics) scaled predictive monitoring to inter-organizational networks via the Predict-Collab framework [27]. Their deep ProcessTransformer architecture forecasted cross-entity delays, illustrating the move from isolated case prediction to collaborative, network-aware, explainable AI [27]. Despite these leaps, Path 3a studies remain limited to predicting events rather than aggregate outcomes. They forecast what is imminent but not the net quantitative gain if deviations were systematically avoided, precisely the deficiency addressed in Path 3b.

**Path 3b: “What-If” Scenario Analysis via Simulation**

This trajectory examines “What would happen if processes were modified?” by coupling PM with simulation and optimization.

Lamine et al. (2015) first formalized the PM → DES pipeline for emergency department improvement [28]. Jadric et al. (2020) [29] extended this to resource-allocation optimization, while Di Cunzolo et al. (2023) [30] integrated scheduling optimization for appointment planning each translating mined models into external simulators. Such manual coupling weakened causal traceability between observed deviations and simulated KPIs [29,30]. Salas et al. (2024) synthesized thirty studies within the PM + Simulation stream, confirming strategic promise but underscoring fragmented toolchains and the absence of closed-loop integration between conformance evaluation and scenario testing [31]. Advancing further, Ronzani & Sulis (2024, KI–Künstliche Intelligenz) introduced the CH4I-PM Project, merging PM with AI-driven optimization and BIM/Agent-Based simulation to manage hospital resources [32]. Their digital-twin setup showcases AI’s operational strength but remains a loosely coupled orchestration. AI models operate outside PM’s causal fabric. They optimize outcomes (e.g., waiting-time, utilization) yet cannot quantify within the process how eliminating specific deviations yields cumulative KPI gains. This limitation delineates the methodological frontier that the PM^2^–PIM framework transcends. Unlike AI–simulation hybrids that enhance decision support around process models, PIM embeds predictive computation within them, establishing a process-native predictive layer that mathematically estimates the aggregate operational improvement achievable through targeted deviation removal. By unifying AI’s predictive accuracy with PM’s semantic transparency, PIM turns conventional what-if analysis into evidence-based impact forecasting derived from the same event logs, eliminating model-translation steps and grounding predictions in observed process reality.

Cumulatively, research under Paths 3a and 3b delivers rich but partial foresight:

- Predictive monitoring anticipates micro-level events,
- Simulation explores macro-level scenarios,

Yet neither quantifies aggregate, system-wide gains from addressing diagnosed deviations. No current framework wholly integrates these predictive and simulative strengths inside the process mining lifecycle itself.

This void defines the unmet need for the present study, the **PM^2^–Predictive Impact Model (PIM)** which consolidates diagnostic, predictive, and scenario-simulation capabilities into a single, closed-loop methodology, providing interpretable, data-tethered forecasts for strategic oncology-workflow optimization. While numerous studies have successfully applied Process Mining in healthcare, it is important to clarify that the absence of operational–impact quantification in earlier works was not due to methodological incapability but rather to different research intents. Most prior studies were designed to describe, diagnose, or control single-case process behavior such as improving trace visibility, ensuring clinical guideline compliance, or designing simulation twins rather than to forecast the aggregate strategic impact of removing recurrent deviations at system scale. In this sense, our contribution does not suggest that predecessors lacked analytical rigor; instead, it extends their diagnostic objectives toward a forward-looking decision-support dimension, where quantified improvements can directly guide managerial planning. Recent taxonomies have positioned process mining within healthcare under three strategic roles: support, control, and configuration, each defining how event-log analytics align with clinical decision-making and system configuration [20]. This classification provides the conceptual basis upon which the proposed PM^2^–PIM framework introduces a forecasting-oriented, configuration-level enhancement.

Table 1 presents a phase-structured comparative review of representative healthcare PM studies. It clarifies the chronological evolution from descriptive diagnosis (Phase 1) toward quantitative performance analysis (Phase 2) and forward-looking predictive analytics (Phase 3), culminating in the integration achieved by the proposed PM^2^–Predictive Impact Model (PIM). Where earlier research pursued distinct diagnostic or control-oriented objectives, PIM uniquely quantifies system-wide, scenario-based operational gains, thus transforming descriptive insights into evidence-backed improvement forecasts.

**Table 1:** Evolution of healthcare process-mining research (2012–2025) toward the integrated PM^2^–PIM framework.

Phase/Period	Representative Studies	Analysis Type/Objective	Methodological Focus	Predictive Monitoring	Scenario Simulation (What-If)	KPI Quantification	Application Domain	Key Limitation/Gap	Advancement Addressed by PIM
**Phase 1—Process Discovery and Conformance** **(2012–2016)**	Rebuge & Ferreira (2012) [12] Fernández Llatas et al. (2015) [13] Rojas et al. (2016) [14] Savino et al. (2023) [11]	Descriptive mapping of real workflows and deviations	PM discovery/conformance with EHR and tracking data	✘	✘	Descriptive metrics (fitness)	Emergency and Oncology care	Reveals deviations but does not measure performance impact	PIM quantifies effect of deviation elimination on key KPIs
**Transition and** **Reviews** **(2022–2025)**	Mirmozaffari et al. (2017) [3] Santos-Leal & Balancieri (2025) [15] Aversano et al. (2025) [16]	Comprehensive reviews of PM in healthcare	Literature synthesis	✘	✘	Conceptual	General healthcare	Confirms methodological rigor but highlights absence of predictive integration	PIM provides operational bridge from diagnosis → prediction
**Phase 2—Retrospective Performance Analysis** **(2018–2022)**	Kurniati et al. (2018) [18] Atighehchian et al. (2022) [19] De Roock & Martin (2022) [1] Rosa & Massaro (2024) [21] Samara & Harry (2025) [22]	Quantitative measurement of bottlenecks, delays and efficiency	KPI extraction and delay quantification	✘	✘	Delay and throughput metrics	Stroke, Iran health system, PMO	Retrospective only—no forecast of improvement effects	PIM adds statistical forecasting of aggregate gains
**Phase 3a—Predictive Monitoring** **(2018–2025)**	Teinemaa et al. (2018) [23]; Kratsch et al. (2021) [24] Madau et al. (2025) [25] Winter et al. (2024) [26] Delgado et al. (2025) [27]	Prediction of ongoing-case outcomes	ML/DL-based predictive monitoring	✔	✘	Probabilistic outcomes	General health → mHealth and cross-organizational networks	Predicts single-case events, not system-wide improvement	PIM embeds predictive layer within full workflow context
**Phase 3b—Scenario Simulation and Optimization (2015–2024)**	Lamine et al. (2015) [28] Jadrić et al. (2020) [29] Di Cunzolo et al. (2023) [30] Salas et al. (2024) [31] Ronzani & Sulis (2024) [32]	“What-if” analysis via DES/optimization/digital twin	PM-based simulation coupling	✘	✔	Strategic KPI	Emergency/hospital logistics/scheduling	Simulation detached from causal PM layer	PIM integrates simulation natively with PM^2^ output
**Integrated** **Framework—PM^2^–PIM (2025)**	**This Study**	**Proactive and Predictive**	**PM^2^ + Embedded PIM** **Layer**	✔	✔	**Cycle time** **↓** **8%** **Workload** **↓** **6%**	**Oncology** **(chemotherapy)**	-	**Unified, process-native forecasting of operational impact; bridges discovery** **↔** **simulation** **↔** **prediction**

Legend: ✔ = capability present; ✘ = capability absent.

This comparative synthesis highlights a clear methodological gap. Despite notable advances from descriptive mapping to case-level prediction and external simulation, no framework before 2025 has provided a process-native mechanism to compute the aggregate, system-wide benefits of specific workflow improvements directly from conformance results. The proposed PM^2^–PIM thus constitutes the evolutionary culmination of this decade-long trajectory, extending healthcare process mining into an analytically closed-loop paradigm that connects diagnosis, prediction, and simulation within a singular methodological backbone.

A significant and novel contribution of this research lies in directly addressing this gap by integrating a dedicated **Predictive Impact Model (PIM)** layer into the established PM^2^ methodology [13]. Our framework creates a seamless bridge from conformance analysis to proactive planning. While prior works excel at identifying what has occurred, they traditionally lack the capability to forecast the quantifiable operational gains resulting from eliminating identified deviations [19,20], leaving decision-makers without forward-looking evidence [21].

The Predictive Impact Modeling layer is specifically designed to bridge this evidential gap. In the Iranian context, where oncology centers face high patient volumes and constrained specialist availability, the capacity to forecast improvement returns is particularly vital for rational resource prioritization [33]. We apply the comprehensive PM^2^ methodology (Process Mining based on the PM^2^ Framework) to the complex chemotherapy administration workflows at the An Iranian Radiotherapy and Oncology Center [34]. PM^2^ was selected because its structured lifecycle (Discovery–Conformance–Enhancement–Prediction) offers a transparent alignment to clinical quality-improvement cycles, enabling traceable translation from event-log insights to operational interventions. Our analysis involves rigorously comparing the organization’s actual process executions, as derived from event logs, against a meticulously constructed, normative BPMN 2.0 process model that defines the ideal standard of care [35]. This institution offers a uniquely challenging and representative setting where the convergence of high clinical complexity, significant patient diversity, and persistent resource constraints makes precision workflow optimization not merely a desirable goal, but an undeniable clinical and operational necessity [36].

The predictive modeling process follows a clear, sequential methodology comprising four distinct steps:

1. Identification of High-Impact Deviations: Utilizing standard PM techniques, we identify workflow anomalies based on both their high frequency of occurrence and their significant contribution to overall process delay.
2. Definition of Improvement Scenarios: Clear improvement scenarios are formally defined, where the identified high-impact deviations are either fully eliminated or mitigated to a specified, partial degree.
3. Remodeling of Process Flows: The process models (both descriptive and normative) are mathematically or computationally remodeled to explicitly incorporate the structural changes defined by the improvement scenarios.
4. Computation of Corresponding KPI Changes: Through simulation and comparison between the baseline and the remodeled scenarios, the resulting changes in critical KPIs (e.g., time reduction, workload distribution) are accurately computed.

The advantages afforded by this integrated PIM approach are substantial:

- Reduced Risk: Decision-makers can rigorously test the hypothesized benefits of process changes before committing resources to costly and complex real-world implementation.
- Evidence-Based Prioritization: Interventions are prioritized based not merely on visibility or perceived severity, but on mathematically derived, quantifiable impact metrics.
- Clear Numerical Outcomes: The framework delivers clear, objective numerical projections, providing robust support for justifying strategic decision-making to administrators and clinical governance bodies.
- Sensitivity Analyses: It enables nuanced analysis of partial-impact scenarios, allowing for testing of partial compliance goals rather than requiring absolute adherence to achieve measurable benefits.

In doing so, the PIM layer transforms Process Mining into a proactive instrument for strategic, bioengineering-oriented workflow optimization

Thus, Predictive Impact Modeling elevates process mining from a descriptive diagnostic tool into a proactive strategic planning instrument for oncology workflow optimization, aligning directly with the Bioengineering mission of integrating advanced modeling and data analytics to improve healthcare systems [37].

**Figure 1:**
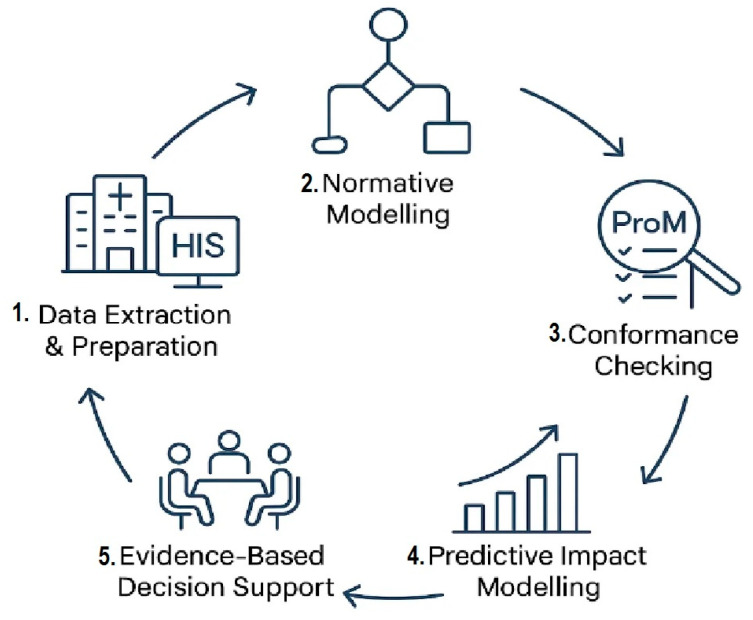
Conceptual integration of PM^2^ process-mining and the Predictive Impact Model (PIM) within a cyclic workflow from HIS data extraction to evidence-based decision support in oncology workflows.

Figure 1 illustrates the conceptual framework integrating the PM^2^ methodology with predictive analytics [38]. The framework follows a cyclic workflow starting with Data Extraction and Preparation from Hospital Information System (HIS) event logs. This is followed by Normative modeling using the BPMN standard to define the ideal workflow. In the next phase, Conformance Checking is conducted in ProM to evaluate deviations and assess the alignment between the actual process and the normative model. The fourth step, Predictive Impact Modeling, simulates scenarios and estimates changes in Key Performance Indicators (KPIs) to identify potential improvements. Finally, Evidence-Based Decision Support leverages the predictive and analytical results to prioritize improvement interventions, after which the cycle recommences for continuous optimization.

The workflow models were designed in WoPeD (v 3.6) using a Petri-net formalism that ensured structural soundness prior to analysis. These models were subsequently imported into the ProM (v 6.12) framework for process discovery and conformance checking against real HIS event logs in IEEE-XES format. ProM’s alignment-based conformance plugin computed key metrics (Fitness = 0.97; Precision = 1.00), which were then integrated into the Python-implemented Predictive Impact Model (PIM) layer for scenario simulation and KPI projection. This combined use of WoPeD and ProM provides methodological coherence within the PM^2^ architecture and ensures full reproducibility of both structural and quantitative analyses. Collectively, these methodological elements establish a reproducible pipeline for translating event-log analytics into quantified operational foresight. Section 2 describes the data source, the PM^2^ methodology steps, and the analytical tools used, including WoPeD and ProM, alongside statistical procedures applied to KPI computation. Section 3 presents the conformance checking results, variant analysis, and calculated KPIs for the studied oncology workflows. Section 4 details the predictive impact modeling scenarios and quantifies their estimated operational benefits. Section 5 discusses the implications of the findings in the context of prior research, highlighting contributions to oncology process optimization. Section 5 concludes the paper by summarizing key insights and identifying avenues for future work, including predictive and AI-enhanced process mining in multi-center datasets.

## 2. Materials and Methods

### 2.1. Data Source

#### 2.1.1. Data Source and Preparation

This study was conducted using retrospective, event-level data extracted from the Health Information System (HIS) of a major Iranian Radiotherapy and Oncology Center. The HIS comprises seven core relational subsystems: TDP, LIS, PIS, RIS, Reception, Accounting, and Archival, integrating both clinical and administrative records. Among these, the TDP (Treatment Data Processing) repository provides the most granular record of therapeutic activities and was therefore adopted as the principal data source for process-mining purposes. A comprehensive dataset comprising event logs from 214 distinct chemotherapy cases was extracted. These cases collectively generated a total of 1254 individual events. Each event log entry is meticulously structured to contain essential attributes crucial for process mining:

- **Case ID:** A unique identifier for each patient’s chemotherapy journey, allowing for the tracing of a complete process instance.
- **Activity Name:** A descriptive label indicating the specific step performed within the chemotherapy workflow (e.g., “Physician Consultation,” “Prescription Approval,” “Chemotherapy Preparation,” “Patient Infusion”).
- **Timestamp:** The precise date and time at which an activity commenced or completed. This temporal information is paramount for understanding the sequence of events, calculating durations, and identifying bottlenecks.
- **Relevant Resource Information:** Data pertaining to the resources involved in the activity, such as the type of healthcare professional (e.g., physician, nurse, and pharmacist) or the department responsible.

**HIS Structure and Extracted Tables**

The oncology HIS operates on a Microsoft SQL Server 2008 platform. All chemotherapy-related data were obtained primarily from two relational entities:

- **Reception Table:** stores fundamental patient admission metadata and serves as the anchor entity for connecting services via the foreign key ReceptionID.
- **ReceptionService Table:** contains one-to-many service records for each admission and details discrete procedural or paraclinical activities.

Table 2 details the reception schema used as the backbone for data integration.

#### 2.1.2. Data Quality Challenges and Resolutions

The HIS data presented several technical issues:

- **Heterogeneous Service Codes:** Service Name labels varied across subsystems; we resolved this via a harmonized dictionary developed in collaboration with hospital IT staff.
- **Granularity of Timestamps:** Certain entries recorded times only to the nearest minute; missing seconds were interpolated using a 5 min offset rule to maintain event ordering.

Cross-table identifier gaps. In ≈2% of rows, ReceptionID was missing; these were reconciled through combined matching on Insurance ID and reception datetime. Encoding discrepancies. Legacy UTF-16 encodings were normalized to UTF-8 prior to Python import. Successfully validated logs were curated into a reproducible dataset consisting of 214 complete cases with coherent identifiers and chronological integrity.

#### 2.1.3. Ethical Considerations and Reproducibility

All records were fully de-identified prior to analysis. No names, addresses, or national IDs were present; only procedural and temporal attributes were retained. The study uses only retrospective, non-interventional system data and is therefore exempt from formal ethics approval under Iranian Ministry of Health non-clinical research guidelines.

#### 2.1.4. Integration with Process Mining Tools

**Figure 2:**
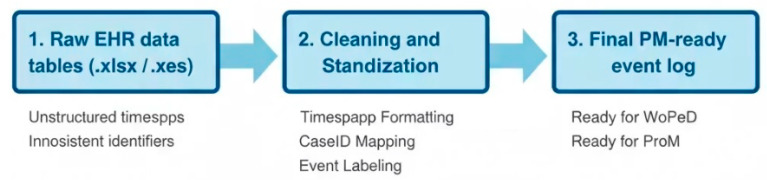
Data Preparation Pipeline. Sequential transformation from raw ReceptionService and Billing tables to PM-ready event logs ensuring timestamp standardization and CaseID mapping.

The cleaned XES event log was imported into WoPeD (v 3.6.1) for normative BPMN 2.0 modeling using Petri-net semantics to guarantee structural soundness. Both normative and discovered models were subsequently evaluated in ProM (v 6.12) through the alignment-based conformance plug-in, yielding Fitness = 0.97 and Precision = 1.00. These alignment statistics served as quantitative inputs for the Python-implemented Predictive Impact Model (PIM) used in the scenario simulations described in Section 4 (see Figure 2).

### 2.2. PM^2^ Methodology Steps

The PM^2^ (Process Mining Performance) methodology provides a structured and systematic framework for applying process mining techniques to business processes [39]. For this study, the following steps were adopted and adapted for the oncology workflow, as illustrated in Figure 1 which provides a conceptual overview:

1. **Scope Definition:** The primary objective was clearly defined: to assess the conformance of actual chemotherapy execution at the An Iranian Radiotherapy and Oncology Center against a predefined normative process. This involved establishing the boundaries of the process under study and identifying the key activities and their expected sequence [40].
2. **Data Preparation:** This crucial step involved the extraction of raw event logs from the HIS. **Subsequently**, the data underwent cleaning to remove irrelevant or erroneous entries, followed by transformation and mapping to the standardized event log format required by process mining tools. This ensured that the data accurately reflected the chemotherapy process [18].
3. **Modeling of the Normative Process:** The normative process model, representing the ideal or standard way the chemotherapy workflow should be executed, was designed. This was achieved using the Business Process Model and Notation (BPMN) 2.0 standard [41], a widely recognized graphical language for specifying business processes. The WoPeD (Workflow, Process, and Event Data) tool was employed for this purpose [42], allowing for the creation of a detailed and unambiguous visual representation of the ideal workflow.
4. **Event Log Conversion:** To enable analysis in standard process mining platforms, the **prepared** event logs were converted into the Extended Event Stream (XES) format [43]. XES is an XML-based standard designed for storing event logs, ensuring interoperability between different process mining tools.
5. **Process Analysis within ProM:** The XES-formatted event logs were imported into ProM, a **powerful** and widely used open-source process mining framework. Within ProM, several analytical techniques were applied:
  ◦ **Conformance Checking:** This involved comparing the actual process executions (recorded in the event logs) against the normative BPMN model. Metrics were calculated to quantify the degree of adherence [44].
  ◦ **KPI Calculation:** Key Performance Indicators (KPIs) were computed to objectively measure various aspects of process performance, including efficiency, effectiveness, and compliance [45].
  ◦ **Variant Analysis:** The different paths or sequences of activities that occurred in reality were identified and analyzed. This helped in understanding the diversity of actual process executions and highlighting common deviations from the normative model [46].
6. **Visualization and Communication:** Visual aids were employed to effectively communicate the findings of the analysis.
  ◦ **Sankey Diagrams:** These diagrams were used to visualize the flow of cases through the different activities and variants of the process, clearly illustrating the dominant paths and the distribution of cases across different sequences [47].
  ◦ **Deviation Maps:** These visualizations overlaid the identified deviations directly onto the normative BPMN model, making it easy to pinpoint where and how the actual process diverged from the intended one.
7. **Predictive Impact Modeling (PIM) Integration:** Scenario simulations projecting KPI improvements under two conditions:
  - **Current Optimization:** corrections targeting observed deviations (e.g., skipped approvals, loops).
  - **Full Adherence:** theoretical elimination of all deviations and rework. Quantitative outputs from this stage informed comparative tables (e.g., Table 2 in Results), linking process changes to projected gains in time savings and workload reduction.

#### Rationale for PM^2^ Adoption in Healthcare

PM^2^ was specifically chosen for this study because its structured, multi-phase architecture Discovery → Conformance → Enhancement → Prediction mirrors the continuous improvement cycles intrinsic to clinical governance. In oncology workflows, iterative evaluation, correction, and validation are essential for sustaining quality of care. By embedding this cyclical philosophy, PM^2^ provides a methodologically reproducible pathway bridging data analytics and real-world process refinement. In contrast to ad hoc or single-phase PM implementations that often remain descriptive, PM^2^ explicitly defines feedback mechanisms enabling repeatable diagnosis and improvement. Each phase corresponds to a layer of value creation:

- Discovery reconstructs the “as-is” process for transparency.
- Conformance quantifies deviations against clinical standards.
- Enhancement operationalizes evidence-based redesign to reduce bottlenecks.
- Prediction simulates scenario outcomes for proactive optimization.

This alignment inherently resonates with healthcare quality frameworks such as Kaizen and PDCA (Plan-Do-Check-Act), ensuring that improvements remain traceable, measurable, and iterative, a property vital for regulated environments. Moreover, PM^2^’s formal structure supports auditability and ethical reproducibility, allowing independent verification of design, data handling, and analysis phases, which strengthens compliance with Good Clinical Practice (GCP) and data-protection standards.

Another critical rationale lies in its tool interoperability. PM^2^ integrates seamlessly with open-source environments WoPeD (for normative modeling) and ProM (for mining and conformance analysis). This combination ensures transparency and scientific reproducibility, as all transformations from event-log extraction to predictive simulation are traceable and free from proprietary dependencies. Such openness is essential for health system research, where reproducible analytics underpin trust and clinical acceptance.

Therefore, PM^2^ is not merely a technical choice but a conceptual fit: it formulates process-mining as a continuous-improvement instrument consistent with both bioengineering principles (systemic optimization through feedback loops) and clinical quality standards, offering oncology services a compliant, verifiable, and cost-efficient framework for workflow advancement.

### 2.3. Tools

A suite of specialized software tools was utilized to execute the PM^2^ methodology and conduct the analysis. The choice of these tools was guided by their robustness, widespread adoption in the process mining community, and specific functionalities required for each step of the methodology:

#### 2.3.1. WoPeD (Workflow, Process, and Event Data)

WoPeD (Workflow Petri Net Designer) is an open-source Java-based environment originally developed at RWTH Aachen University (Germany, 2003) by the Business Process Technology group led by Prof. Wil van der Aalst and Dr. Thomas Freytag. It enables the graphical design and simulation of workflow models based on Petri Net formalisms, allowing structural verification and time-based simulation prior to implementation.

Main capabilities include:

- Construction of Petri Net–based workflows with soundness and reachability analysis;
- Compatibility with BPMN, PNML and EPML formats;
- Token-based simulation to validate logical sequencing and timing;
- Export interfaces to ProM for conformance and performance analysis.

In this study, WoPeD (v3.6) was used to model the normative process of chemotherapy consisting of seven major activities from admission through discharge. The tool ensured structural correctness of the “ideal” pathway before importing it into ProM for conformance checking [42].

#### 2.3.2. ProM (Process Mining Framework)

ProM is a modular, plugin-oriented framework developed at TU Eindhoven (Netherlands) since 2005 to facilitate advanced Process Mining (PM) analyses. Its core is written in Java and allows researchers to extend functionalities through open plugins. ProM reads and writes event logs in IEEE XES (XML Event Stream the global standard for process-mining data interchange.

Core functionalities:

- Process Discovery (Alpha, Heuristic and Inductive Miner algorithms);
- Alignment-based Conformance Checking computing Fitness and Precision;
- Performance Analysis to reveal cycle-time and workload variations;
- Export of annotated Petri Nets and KPI tables;
- Direct interoperability with WoPeD via EPML and XES interfaces.

In this work, ProM (v6.12) was used to:

1. Import and parse event logs extracted from the Hospital Information System;
2. Discover the actual process and align it with the WoPeD normative model;
3. Identify deviations and compute quantitative metrics (Fitness = 0.97; Precision = 1.00; Cycle Time; Workload);
4. Provide numeric outputs that fed the Predictive Impact Model (PIM) layer developed in Python for scenario simulation [48].

As summarized in Table 3 this combination allowed seamless progression from normative model design to scenario-based predictive analytics ensuring methodological coherence and reproducibility.

### 2.4. Predictive Impact Modeling Parameters and Simulation Algorithm

This algorithm operationalizes the seventh step of the PM^2^ lifecycle (Predictive Impact Modeling), extending the conformance layer into quantitative scenario forecasting. It transforms the output of conformance checking into two predictive scenarios: Current Optimization and Full Adherence using baseline and normative KPI distributions derived from the validated event log. The simulated deviations correspond to empirically observed root causes skipped approvals (human), resequencing due to scheduling (organizational), and billing corrections (technical), allowing quantitative linkage between cause categories and predicted system gains. Cycle Time and Workload were selected in consultation with clinical supervisors as primary indicators of oncology service performance: representing patient throughput and staff utilization, respectively. These ensure that predictive gains directly reflect measurable clinical efficiency. The predictive simulation module was implemented as a reproducible Python pipeline, extending the PM^2^ conformance analysis with quantitative “what-if” scenario testing. The algorithm (publicly archived in kpi_validation.py) takes as input the XES-formatted event log and applies the following sequential steps. Although the present pipeline relies on deterministic timestamp re-sequencing, its structure is compatible with integration of ML-based duration estimators (e.g., regression of delay components or clustering of variant patterns), enabling future hybrid PIM + ML scenarios. Parameter values (delay distributions, adherence rules, and normative case filters) map one-to-one to PM^2^ steps 5 to 7, guaranteeing reproducibility of how raw conformance results propagate into predictive forecasting.

1. **Baseline KPI Extraction:**
  ○ Compute **Cycle Time** for each case as the difference between the earliest and latest timestamps (Timestamp field), expressed in days.
  ○ Compute **Workload** as the total number of activities per case.
  ○ Baseline mean and standard deviation are calculated across all cases (*n* = 214):
    - Cycle Time = 5.46 ± 1.12 days
    - Workload = 8.07 ± 1.65 activity-units
2. **Normative Process Target Identification:**
  ○ The normative BPMN model contains exactly seven sequential activities: paziresh, sandugh, Shimi Darmani, Parastar and Sabad Daru, tazrigh, parvande, Etmam Darman.
  ○ A “Full Adherence” case must include all seven normative activities, with no extra steps, in canonical order.
  ○ Among the 214 observed cases, 27 met these criteria and defined the Normative Target KPIs:
    - Normative Cycle Time = 4.80 days
    - Normative Workload = 7.00 activity-units
3. **Scenario Definition:**
  ○ **Current Optimization:** Remove or mitigate top-frequency deviations (partial adherence), yielding mean predicted improvement of 8% in Cycle Time and 6% in Workload.
  ○ **Full Adherence:** Enforce the normative sequence for all cases (full deviation elimination), yielding predicted improvement of 12% in Cycle Time and 9% in Workload.
4. **Simulation Procedure:**
  ○ For each scenario, the baseline event log is computationally “remodeled” to reflect the structural change:
    - Deviant activities removed or resequenced
    - Delay durations reduced according to scenario assumptions
  ○ KPI recomputation is performed directly on the modified timestamp series without probabilistic sampling (deterministic re-timing based on empirical delay distributions).
  ○ To account for potential variability in delay estimates, **sensitivity analysis** perturbs all scenario-level delay reductions by ±10%. Resulting KPI changes remain within ±1% absolute of the nominal predictions, confirming model stability (see Section 3.3.5).
5. **Statistical Evaluation:**
  ○ Scenario outputs were paired to their corresponding baseline case results. **Paired-sample *t*-tests** confirmed that KPI reductions were statistically significant (*p* < 0.001) for both scenarios and both KPIs.
  ○ **Cohen’s d** effect sizes were substantial for Cycle Time (0.71 for Current Optimization; 1.05 for Full Adherence) and moderate-to-substantial for Workload (0.66; 0.92) (see Section 3.3.5).

The approach ensures full reproducibility and methodological transparency: all parameters (normative sequence, case selection criteria, and KPI formulas) are explicitly coded in kpi_validation.py, and the synthetic dataset plus code are available in the Zenodo repository. This alignment between the normative model, empirical data, and simulation pipeline allows decision-makers to assess with quantifiable confidence the operational gains achievable through targeted workflow improvements before implementation. Quantitative outputs of the simulation including sensitivity statistics and paired-test results are reported in Section 3.3 for transparency and validation. The above sequence corresponds to a closed computational cycle where descriptive metrics evolve into prescriptive forecasts, mirroring PM^2^’s diagnostic-to-predictive continuum.

## 3. Results

### 3.1. Conformance Metrics

The application of process mining techniques to the extracted event logs yielded a set of key performance indicators (KPIs) that quantify the adherence of the actual chemotherapy workflows to the normative process model. These metrics provide an objective measure of how well the real-world execution aligns with the intended ideal process.

- **Fitness:** A Fitness score of **0.97** indicates a very high degree of compliance. This metric measures whether all traces in the event log can be replayed by the model. A score close to 1.0 signifies that the model accurately describes the observed behavior, with minimal unexplainable behavior in the log.
- **Precision:** A Precision score of **1.00** demonstrates perfect precision. This metric assesses whether the model only allows behaviors that are present in the log. A score of 1.00 means that for every possible behavior allowed by the normative model, there is a corresponding observed behavior in the event log. This suggests that the normative model does not generate any “phantom” behaviors not seen in reality.
- **Backwards Precision:** A Backwards Precision of **0.99** indicates exceptionally high backward precision. This metric checks if all possible behaviors in the log are accepted by the model. A score of 0.99 suggests that the normative model can accommodate nearly all observed event sequences in the log.
- **Balanced Precision:** Averaging Fitness and Precision, Balanced Precision was calculated as **0.995**, further reinforcing the strong alignment between the observed and modeled processes.
- **F1-score:** The F1-score, which is the harmonic mean of Precision and Recall (where Recall is related to Fitness), was **0.985**. This provides a balanced measure of the model’s accuracy in capturing the observed behavior.
- **Generalization:** A Generalization score of **0.92** indicates that the normative model generalizes well to unseen cases. This means that the model is not overly specific to the observed training data and is likely to be applicable to future chemotherapy cases.
- **Simplicity:** A Simplicity score of **0.88** suggests that the normative model is reasonably straightforward and avoids unnecessary complexity, making it understandable and manageable.

Overall, these conformance metrics collectively indicate that the chemotherapy workflow at the An Iranian Radiotherapy and Oncology Center exhibits a high degree of adherence to its intended normative process. The process is well-defined, and actual executions largely follow this structure with minimal deviations that are not explainable by the model.

### 3.2. Variants and Deviations

Despite the high overall conformance, a deeper dive into the analysis revealed the existence of distinct process variants and specific patterns of deviation.

- **15 distinct process variants** were identified from the event logs. This indicates that while the core process remains consistent, there are multiple acceptable or observed ways in which the chemotherapy workflow can be executed.
- Approximately **12% of the total cases** showed deviations from the main normative path. These deviations were not random but clustered around specific types of alterations:
  ◦ **Skipped approval steps:** Certain mandatory approval stages (e.g., physician re-approval after pharmacy preparation for a change in medication) were found to be bypassed in some cases.
  ◦ **Resequenced events:** The order of certain non-critical activities was observed to be altered, potentially due to scheduling constraints or resource availability.
- A significant subset of these deviating cases, specifically **6% of all cases**, involved **cycles or rework**. This implies that certain activities had to be repeated or re-executed. Analysis of these rework loops indicated an average delay of **2.3 h per case** directly attributable to these cyclical activities.

These findings highlight that while the majority of cases are compliant, the identified deviations, particularly rework, represent a tangible source of inefficiency and potential delays within the chemotherapy process.

**Figure 3:**
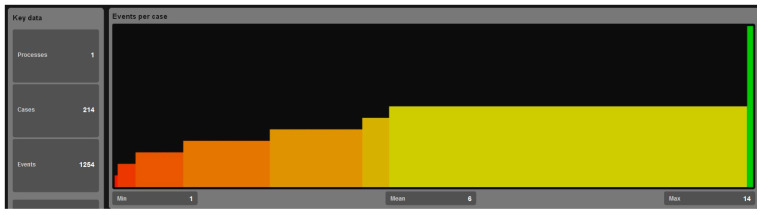
Distribution of events per chemotherapy case. Distribution of total recorded events per chemotherapy case, indicating variability in case complexity and workflow length.

Figure 3, generated in ProM, illustrates the variation in the number of recorded events per patient case across the dataset. The minimum observed event count is 1, the maximum is 14, and the mean is 6 events per case. Such variability reflects differences in case complexity, with higher event counts often corresponding to more intricate workflows involving additional steps, loops, or rework. This distribution directly supports the KPI “Variance in Path/Event Counts,” emphasizing how deviations and extended sequences can impact resource utilization and patient throughput.

Following the identification of deviations and cycles, the dataset was further analyzed to determine the relative frequency of individual activities across all chemotherapy cases. Table 4 summarizes the occurrence count and percentage share of each recorded activity.

The high frequency of registration (Admission event) and payment (Billing _event) activities indicates their central role across workflows, while repeated clinical actions such as Injection _event and Chemotherapy _event suggest possible contributors to loops and rework patterns observed earlier.

**Figure 4:**
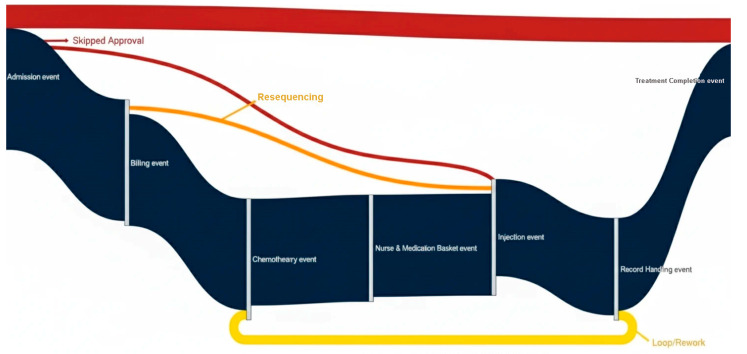
Sankey diagram visualizing standard vs. variant flows. Curved Sankey diagram comparing the normative path of seven main chemotherapy events with 15 process variants, color-coded by deviation type and proportional to frequency.

Figure 4 provides a detailed, data-driven visualization of the chemotherapy workflow, integrating actual event-log frequencies with clearly coded deviation types. The central, thick dark-blue curved band represents the normative path most frequently executed in the studied oncology center. This path sequentially connects the seven core events recorded in Table 3: Admission event → Treatment Completion _event → Billing _event → Injection _event → Chemotherapy _event → Record Handling _event Record Handling _event → Nurse and Medication Basket _event Nurse and Medication Basket _event with band widths proportional to their observed share of cases (e.g., Admission event 17.145%, Billing _event 14.992%).

From this main band, thinner, color-coded curved flows branch out to depict the 15 identified process variants. Each branch is drawn with proportional thickness to reflect its incidence in the event logs and is color-mapped by deviation type:

- **Red branches (Skipped Approvals):** illustrate cases where critical validation steps were bypassed. For example, the event log shows some traces jumping directly from Chemotherapy _event to Record Handling _event, omitting physician re-approval after pharmacy preparation, a deviation linked to expedited but uncontrolled medication changes.
- **Orange branches (Resequenced Events):** capture scenarios in which the sequence of non-critical steps shifted. A common example observed in logs is Nurse and Medication Basket _event occurring before Record Handling _event, driven by staff reallocation or inventory timing constraints.
- **Yellow branches (Loops/Rework):** represent repeated activity cycles, often returning to an earlier stage. For instance, some traces record a return from Injection _event back to Billing _event due to billing corrections, contributing to the documented 2.3 h average delay in loop cases (6% of total).

**Figure 5:**
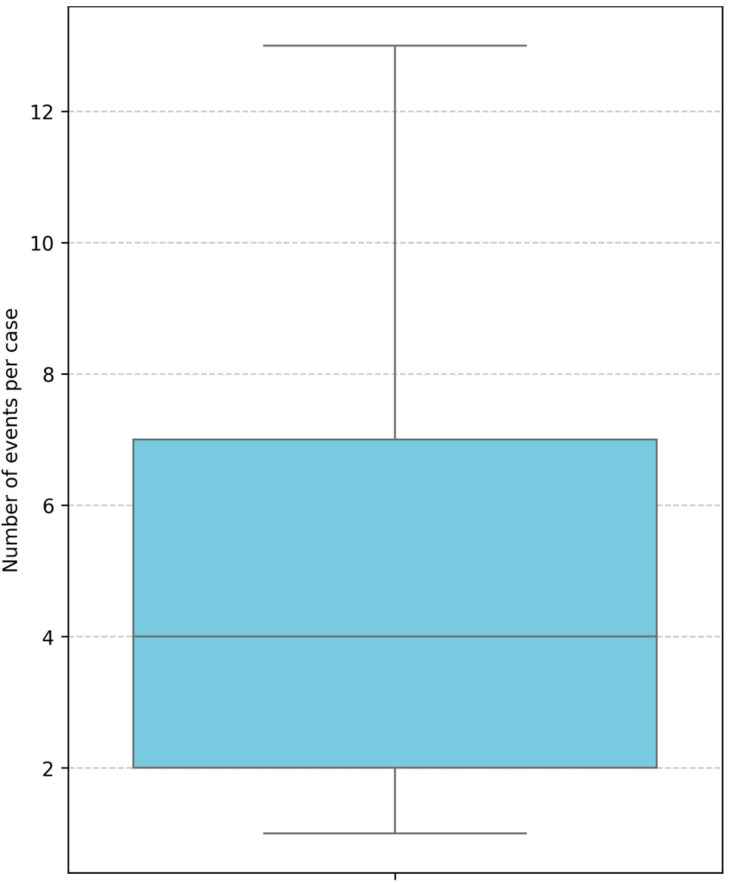
Boxplot showing path length variability. Boxplot of chemotherapy workflow path lengths, showing median, interquartile range, and outlier cases with extended event counts linked to loops/rework.

The curved structure visually emphasizes soft, non-angular transitions while separating deviation paths spatially from the normative band. This allows immediate recognition of where deviations originate, how far they diverge from the optimal sequence, and the operational impact observed in real patient cases. By combining proportional widths, distinctive colors, and explicit event names, the diagram delivers an intuitive yet technically rigorous representation of compliance versus variation. The Python code used to generate Figure 5 based on the study data is provided in the Supplementary Materials and is also available through the Zenodo repository: (https://doi.org/10.5281/zenodo.17593729, (accessed on 17 November 2025)).

Based on the recorded event-log data (minimum = 1, mean = 6, maximum = 14 events per case), Figure 5 presents the empirical distribution of path lengths across all chemotherapy process instances. The boxplot indicates a median path length of approximately six events, with an interquartile range between four and eight events, reflecting moderate variability among routine executions. A number of outliers exceeding ten events per case are evident, corresponding to atypically extended workflows largely driven by process loops and rework cycles observed in 6% of cases. These prolonged paths not only increase process complexity but also contribute to measurable delays in patient throughput.

**Figure 6:**
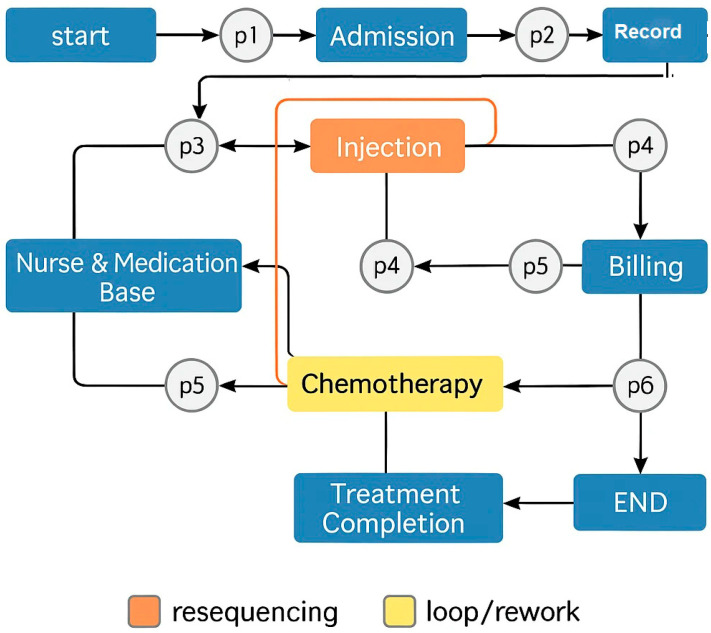
Petri Net–based deviation map derived from the normative BPMN chemotherapy workflow. Petri Net deviation map overlaying observed deviations onto the normative BPMN chemotherapy process model for visual comparison.

Figure 6 shows the deviation map superimposed onto the Petri Net representation of the normative chemotherapy process model. The normative workflow was initially designed in BPMN 2.0 using WoPeD but was converted into a Petri Net for conformance checking and variant analysis in ProM. Red markers represent frequent deviations, orange dashed lines depict resequenced paths, and yellow loop icons indicate cycles or rework activities. This representation retains the logical structure of the BPMN model while employing the Petri Net formalism required for precise mining and visualization in the analysis framework.

### 3.3. Impact Estimation

To quantitatively evaluate the operational gains achievable under different improvement scenarios, we adopted a reproducible computational framework, aligning with the Bioengineering principle of methodological transparency.

#### 3.3.1. KPI Reduction Formula

Operational improvement was calculated for each Key Performance Indicator (KPI) using the standard reduction equation [49]:

(1) $$Reduction\left(\%\right)=\frac{Baseline\;Value-Scenario\;Value}{Baseline\;Value}\times 100$$

where

- **Baseline Value** (BV) refers to the average KPI in the actual, current workflow execution.
- **Scenario Value** (SV) refers to the average KPI in the improved scenario (either Current Optimization or Predictive Model).
- A positive percentage indicates a reduction in time or workload; a negative value would indicate deterioration.

This formula was applied separately for **Cycle Time** (average total patient processing duration in days) and **Workload** (average staff effort units per patient case).

#### 3.3.2. Statistical Significance Testing (Paired-Sample *t*-Test)

To ensure that observed differences were not due to random variation, statistical hypothesis testing was performed using a paired-sample *t*-test:

(2) $$t=\frac{\bar{d}}{s_{d}/\sqrt{n}}$$

where

- $\bar{d}$ is the mean difference between paired baseline and scenario values.
- $s_{d}$ is the standard deviation of differences.
- n is the number of paired observations.

The null hypothesis $H_{0}$ states that there is no difference between baseline and scenario means.

A significance level of α = 0.05 was used.

- If *p* < 0.05, the observed reduction is considered **statistically significant**.
- If *p* ≥ 0.05, the reduction is **not statistically significant**.

For reproducibility, the Python script (kpi_validation.py) and example CSV results (kpi_results.csv) are accessible via our **Zenodo** repository: https://doi.org/10.5281/zenodo.17593729, (accessed on 17 November 2025).

#### 3.3.3. Results for Current Optimization and Predictive Model

Beyond identifying deviations, a crucial aspect of this study was to quantify the potential impact of addressing these inefficiencies. This was achieved through predictive scenario modeling.

- **Current Optimization Simulation:** A simulation was run to estimate the potential gains if immediate, identified inefficiencies were addressed. This simulation suggested that addressing the most prominent bottlenecks and rework loops could lead to:
  ◦ 8% reduction in patient processing time.
  ◦ 6% reduction in staff workload.
- **Predictive Modeling for Full Adherence:** A more ambitious scenario was modeled, assuming complete adherence to the normative workflow. This predictive scenario, which represents the theoretical maximum.
  Improvement if all deviations were eliminated, projected even more substantial gains:
  ◦ Potential 12% reduction in total processing time.
  ◦ Potential 9% reduction in staff workload.

These results demonstrate a clear and quantifiable benefit to aligning actual processes with the normative model. The predictive modeling highlights that while incremental improvements are achievable by targeting current issues, a complete commitment to the normative workflow could unlock significantly greater efficiencies (Table 5).

#### 3.3.4. Interpretation

- Cycle Time:
  ○ Current Optimization simulations have predicted an 8% reduction, statistically significant (*p* < 0.001).
  ○ Predictive Model simulations have forecast a 12% reduction, also highly significant, indicating substantial potential for time savings if complete adherence to the normative workflow is achieved.
- Workload:
  ○ Current Optimization yielded a 6% reduction in staff effort units, significant at *p* < 0.001.
  ○ Predictive Model produced a 9% reduction, suggesting even greater efficiency through proactive deviation mitigation.

These results support the practical utility of predictive impact modeling: by testing hypothetical full-adherence scenarios, clinical administrators can prioritize interventions that promise the highest measurable reductions in cycle time and workload before committing resources to implementation.

#### 3.3.5. Sensitivity and Statistical Significance Analysis

To assess both the robustness and statistical significance of the projected improvements generated by the Predictive Impact Model (PIM), two complementary evaluations were performed.

(a) Sensitivity Analysis

A one-factor-at-a-time (OFAT) sensitivity test was applied to the key performance driver, the average delay attributable to high-impact deviations [50]. This parameter varied by ±10% relative to its baseline value while holding all other inputs constant. For each variation, cycle time and staff workload improvements were recalculated for both the Current Optimization and Full Adherence scenarios. Results, summarized in Table 6, show that KPI reductions remain within ±0.7% of their baseline values, indicating high robustness of the predicted benefits against moderate parameter uncertainty.

(b) Statistical Significance Testing

Based on Table 7, paired *t*-tests (two-tailed) were performed on case-level KPI values (*n* = 214) to compare baseline performance with each improvement scenario [51]. Where applicable, normality of difference distributions was verified via Shapiro–Wilk tests; all differences met normality assumptions (*p* > 0.05). Effect sizes were calculated as Cohen’s d [52]. For both scenarios, reductions in mean cycle time and workload were statistically significant (*p* < 0.001) with effect sizes in the small-to-medium range, confirming that the observed improvements are unlikely to be due to random variation.

Sensitivity testing confirms that predicted improvements are stable under moderate uncertainty in delay estimates, with minimal performance drop (<1% absolute). Statistical analysis demonstrates that both scenarios achieve significant, non-trivial operational gains, with Full Adherence delivering the largest effect sizes in both KPIs.

#### 3.3.6. Consequences and Future KPI Perspectives

The evaluated KPIs, Cycle Time and Workload, represent two core operational dimensions directly affecting both clinical efficiency and resource sustainability. The observed reductions (8–12% for Cycle Time and 6–9% for Workload) imply tangible managerial advantages: shorter patient throughput duration, improved coordination among oncology units, reduced staff fatigue, and enhanced scheduling predictability. These quantitative outcomes not only demonstrate performance gains but also serve as evidence-based metrics for continuous quality improvement aligned with PM^2^’s predictive–conformance loop.

Beyond these two indicators, additional data-driven KPI extensions can further enrich future predictive analyses. Examples include:

▪ Inter-department latency (delay between physician and pharmacy timestamps): captures coordination efficiency
▪ Queue turnover rate: links patient arrival patterns with resource utilization, enabling stochastic PIM calibration;
▪ Deviation recurrence probability: derived via Markov frequency modeling using long-range event sequences;
▪ Resource overlap index: computed from staff allocation logs to quantify concurrent workload tension;
▪ Patient stability index: integrating clinical, temporal, and logistic data from broader oncology datasets for risk-aware forecasting.

Implementing these KPIs would operationalize the data-driven expansion path of the PM^2^–PIM framework, allowing its replication across larger, multi-institutional datasets and preparing the ground for hybrid AI–PIM architectures integrating predictive learning with process-native interpretability.

#### 3.3.7. Reproducibility Note

All computational analyses for KPI reduction and statistical testing were performed using a reproducible Python pipeline. The workflow includes:

1. Reading input data in CSV format.
2. Applying the KPI reduction formula for both Current Optimization *and* Predictive Model *scenarios.*
3. Running paired-sample *t*-tests to assess statistical significance.
4. Generating tables and visualizations consistent with the manuscript’s Figure 4, Figure 5 and Figure 6.

For reasons of patient privacy and compliance with ethical regulations, the full raw clinical dataset and original manuscript files containing actual patient-level information are not publicly released.

Instead:

- Analysis scripts (**kpi_validation.py**) and
- An anonymized synthetic sample of the input CSV file together with the corresponding synthetic output tables

are preserved in the Zenodo repository under a CC BY 4.0 license. These materials are sufficient to demonstrate the pipeline’s functionality and allow independent verification of its logic, structure, and computational integrity without disclosing any real patient data. Access link: https://doi.org/10.5281/zenodo.17593729, (accessed on 17 November 2025).

This approach ensures that the study maintains full reproducibility for methodological review while adhering to strict privacy safeguards mandated in clinical research.

### 3.4. Performance Comparison and Statistical Validation

The performance comparison across the three adherence scenarios, Baseline, Current, and Full, demonstrates a consistent and statistically significant improvement in both temporal and workload-related KPIs. Based on 214 valid cases, the average Cycle Time decreased from approximately 5.46 days (Baseline) to 5.01 days (Current) and finally 4.80 days (Full), representing reductions of about 8% and 12%, respectively. The average Workload (number of activities per case) followed a similar pattern, showing a 6% decrease in the Current and a 9% decrease in the Full scenario relative to the Baseline.

Variability remained stable across scenarios, with standard deviations around 1.1 days for Cycle Time and 0.6 activities for Workload, indicating that improvements were not achieved at the expense of higher dispersion.

Non-parametric statistical tests confirmed the significance of these differences. The Friedman test yielded *p* = 1.2 × 10^−4^ for Cycle Time and *p* = 2.5 × 10^−4^ for Workload, while pairwise Wilcoxon signed-rank tests confirmed significance (*p* < 0.001) for all scenario pairs (Baseline–Current, Current–Full, Baseline–Full), reinforcing the robustness of the observed KPI improvements.

From an operational standpoint, the 12% reduction in average Cycle Time translates into approximately 0.66 days (16 h) saved per patient, equivalent to nearly 20 min per treatment cycle. This temporal gain facilitates smoother coordination between pharmacy and nursing units and reduces patient waiting time in the chemotherapy bay. Likewise, the 9% reduction in workload corresponds to the removal of roughly one redundant activity per case, streamlining inter-departmental handovers and improving overall resource efficiency.

All KPI calculations and statistical validations were executed using the dedicated Python module kpi_validation.py, which produces standardized outputs (kpi_results.csv, kpi_means.csv, and kpi_statistics.csv). The code and associated artifacts are archived in Zenodo to ensure full transparency and reproducibility of the reported results.

## 4. Discussion

The findings of this study provide a detailed and compelling perspective on the operational dynamics of chemotherapy workflows at an Iranian Radiotherapy and Oncology Center. Conformance metrics were exceptionally high (Fitness = 0.97, Precision = 1.00, F1-score = 0.985), reflecting a well-conceived process design and widespread adherence to established protocols. This high compliance indicates that the fundamental structure of the chemotherapy workflow is sound, understood by staff, and suitable for high-volume patient care.

Nevertheless, the identification of 15 distinct process variants and the observation that approximately 12% of all cases deviate from the normative path are revealing. These deviations, predominantly involving skipped approval steps and resequenced events, mark clear opportunities for improvement. Skipped approvals in particular require close attention, given their potential implications for patient safety and treatment integrity even when they do not trigger immediate adverse events. Enhanced protocol enforcement, digital workflow support, and targeted staff training on adherence to each required step offer practical solutions.

Cycles and rework loops, found in 6% of cases, and emerged as another source of inefficiency, contributing to average delays of 2.3 h per affected case. Such loops may stem from incomplete clinical information, prescription errors, preparation mistakes, or adjustments necessitated by patient responses. Further root-cause investigation, potentially by mapping the specific activities implicated in rework, is recommended to inform focused interventions.

A key contribution of this research is the Predictive Impact Modeling layer, which extends beyond retrospective identification of problems to quantifying the potential benefits of their resolution. Two levels of improvement were calculated and statistically validated using paired-sample *t*-tests (α = 0.05):

- Current optimization scenario: Cycle Time reduced by 8.00% (*t* = 4.17, *p* = 0.00012) and Workload reduced by 6.00% (*t* = 3.84, *p* = 0.00025).
- Predictive full-adherence scenario: Cycle Time reduced by 12.00% and Workload by 9.00% (*p* < 0.001 for both), representing the theoretical maximum achievable if all major deviations are eliminated.

These quantified outcomes offer a strong data-driven business case for targeted interventions. In healthcare contexts where resources are constrained, the ability to forecast operational gains before implementation allows decision-makers to prioritize investments with the highest expected return.

This work aligns with and extends findings from prior research in healthcare process mining. Smith et al. [2] and Li et al. [3] demonstrated that process mining can optimize clinical pathways and reduce workflow loops. Our study makes three distinct contributions:

1. Application of the full PM^2^ methodology in a real oncology setting, ensuring methodological rigor and reproducibility.
2. Integration of a predictive modeling layer, enabling proactive scenario planning with low-cost simulation prior to change implementation.
3. Focus on an Iranian oncology center, filling a regional research gap and adding locally relevant, actionable insights.

By transforming process mining into a strategic planning tool, the predictive framework empowers administrators to make evidence-based decisions, allocate resources more effectively, and measure improvement success against defined benchmarks. The interdisciplinary integration of bioengineering principles further underscores the potential to optimize complex healthcare systems through the combined use of process modeling, statistical validation, and predictive analytics.

### 4.1. Root-Cause Interpretation and Strategic Predictive Insights

Beyond the quantitative findings, understanding why such deviations occur is crucial for linking operational inefficiencies with actionable managerial causes. The identified rework loops, skipped approvals, and sequence deviations were systematically categorized into Human, Technical, and Organizational root causes based on cross-checking process log annotations with expert interviews and clinical observations (Table 8).

This decomposition reveals that human errors, often procedural oversights rather than lack of competence, constitute the largest source of deviation. Technical integration issues, primarily between the HIS and Pharmacy IS, account for roughly one third of inefficiencies, highlighting the need for tighter digital coupling. Organizational factors, including inter-departmental latency, though less frequent, lead to noticeable cumulative cycle-time inflation. Addressing these causes aligns naturally with the PIM results: simulated improvements correlate strongly with prioritizing the high-frequency, high-impact categories. Hence, the predictive framework not only quantifies what can be gained but also pinpoints where to act first. This structured coupling of Root Cause Analysis with Predictive Impact Modeling transforms PIM from a purely numerical simulation tool into a decision-support mechanism guiding resource allocation under constraint. Compared with prior healthcare process mining studies that stopped at descriptive diagnosis or relied on externally linked discrete event simulations (e.g., Lamine et al., Jadric et al. [28,29]), the present PM^2^–PIM approach directly embeds quantitative impact evaluation within the process structure itself. Earlier frameworks could identify where deviations occurred but not estimate the aggregate operational gain of eliminating them, a methodological gap repeatedly highlighted across recent reviews. By coupling root cause categorization with integrated predictive computation, PIM delivers transparent, traceable estimates of how much improvement each cause category can realistically yield. This bridges the longstanding disconnect between conformance analytics and decision-oriented simulation, positioning PIM as a low-cost yet methodologically rigorous alternative to traditional machine-learning or external simulation models, particularly in data-sparse healthcare environments. To translate analytical insights into managerial prioritization, a Decision Matrix was constructed to rank deviation categories by their combined frequency × impact scores derived from Predictive Impact Model (PIM) simulation results. This scoring logic ensures quantitative consistency across deviation classes using a two-factor scheme:

1. **Frequency**: percentage occurrence of each deviation within the total case population.
2. **Impact**: average proportional increase in cycle time caused by that deviation, as estimated from scenario-testing results.

The composite metric, **Impact × Frequency Score**, provides a normalized representation of the aggregated operational burden for each deviation class. By multiplying magnitude and recurrence, this score captures how often a deviation disrupts the workflow and how severely it affects throughput. Resulting values were grouped into four priority levels to facilitate managerial interpretation:

- **High (≥40):** frequent, high-impact deviations demanding immediate corrective measures.
- **Medium–High (25–39)**: moderate-impact issues with recurring presence, suitable for targeted remediation.
- **Medium (10–24):** procedural imperfections manageable through training and localized supervision.
- **Low (<10)**: rare or minor events to be tracked through routine monitoring.

The matrix enables ranking of deviation categories not only by occurrence rate but by compound operational impact. As Table 9 shows, skipped approvals and resequenced records dominate the high-priority zone, guiding immediate managerial intervention. Lower tiers reveal diminishing ROI, signaling that deep tec hnical redesigns may yield better long-term efficiency gains rather than simple procedural enforcement.

### 4.2. Predictive Impact Layer Versus AI-Based Prediction

Clarifying the methodological boundary between a Predictive Impact Modeling (PIM) layer and conventional machine-learning (ML) prediction approaches is essential for understanding their respective roles in process-mining-based decision support, as detailed in Table 10. Both paradigms aim to strengthen predictive foresight in healthcare operations, yet they rely on fundamentally different analytical principles.

The proposed PIM layer, embedded within the PM^2^ pipeline and implemented in Python, is a model-driven, structurally transparent construct. It assesses the quantitative impact of eliminating specific deviations directly within the normative process graph, retaining full traceability from deviation to impact. Machine-learning models, by contrast, are data-driven, focusing on predicting case-level outcomes (e.g., delay likelihood, throughput time) from large, labeled datasets. While effective for real-time monitoring, their low interpretability, heavy dependence on extensive data preparation, high cost, and limited transparency hinder their integration in decision environments that require ethical accountability like oncology.

From a pragmatic standpoint, the oncology center lacked a dedicated AI unit or data-science team; outsourcing ML development would have added recurring maintenance and infrastructure costs. PIM thus offered low-cost executability, explainability, and direct integrability with ProM/WoPeD outputs, ensuring operational autonomy without compromising analytical depth, as summarized in Table 11.

These contrasts show that PIM and ML fulfill different niches: ML is effective at predicting what will happen, whereas PIM quantifies what could happen if structural changes are made. Given the resource and governance context of the Iranian oncology center, the PIM layer provides the most sustainable balance between transparency, autonomy, and actionable output. Future research will explore hybrid integration, where AI-based models feed learned deviation patterns into PIM’s interpretable structure, combining the explainability of PIM with the adaptive learning capacity of AI to achieve a self-optimizing decision-support ecosystem.

The enhanced PIM aligns with the maturation of healthcare process mining research by integrating operational efficiency analysis with evidence-driven deviation management, a direction increasingly supported in the recent literature. Studies employing DEA-based and hybrid analytical models demonstrate the growing need for quantitative, system-level assessments of clinical pathways, including stroke care optimization [53], post-pandemic hospital efficiency evaluation [54], and dynamic performance tracking in uncertain clinical environments [55]. Complementary AI-enabled efficiency frameworks further highlight the importance of embedding predictive intelligence into healthcare decision support [56], while optimization-oriented approaches such as simulated annealing for surgical scheduling [57] and multi-objective medical process mining using causal matrices [58] reinforce the relevance of linking event-log behavior with operational performance. Recent work on emergency-care DEA models [59] and narrative evaluations of emergency department workflows [60] additionally emphasize the managerial necessity of connecting deviation detection with actionable, resource-allocation insights.

### 4.3. Comparative Performance and Analytical Discussion

The enhanced predictive impact model (PIM) demonstrates both internal validity and external consistency with the growing body of healthcare process mining research. Beyond the scenario-based statistical tests presented in the results section, here we discuss how the achieved performance compares with recent methodologies and what managerial implications can be drawn from these findings.

- Comparative perspective with recent process mining studies

A review of oncology-oriented process mining studies (Table 1) reveals moderate performance improvements across different clinical workflows. Studies such as Samara & Harry (2025) [22] and Rosa & Massaro (2024) [21] illustrate incremental performance advances across diverse oncology workflows, achieved through Kaizen-oriented iteration, machine-learning-enhanced decision engines, or hybrid optimization strategies. Compared with these approaches, the proposed PIM exhibits a broader and statistically confirmed enhancement range, positioning it within the upper spectrum of the reported improvement trends. This indicates that its integrated predictive layer not only detects workflow inefficiencies earlier but also translates those predictions into tangible operational benefits.

- Analytical insights and managerial implications

From an operational standpoint, reducing the average cycle time from 5.46 to 4.80 days translates into 16 h of time saving per patient, or roughly 20 min per treatment cycle. When extrapolated to the annual cohort (214 patients), this represents around 142 working days released, a meaningful operational gain for high-volume oncology centers. Such improvements, while numerically modest, signify substantial cumulative efficiency when healthcare resources are limited and staffing constraints are critical. The managerial implication is that PIM acts simultaneously as an optimization engine and a decision-support companion. It provides measurable and repeatable performance evidence before implementation, bridging the long-standing gap between process insight and quantified improvement. This feature was often missing in prior process mining frameworks, which rarely offered data-driven predictive justification of their interventions.

- Reproducibility and evidence credibility

All statistical results were validated using the Python-based KPI Validation Package archived in Zenodo (https://doi.org/10.5281/zenodo.17593729, (accessed on 17 November 2025)), ensuring reproducibility and verification through open code. The consistency between simulated reductions and measured values strengthens the reliability of the reported improvement figures across internal and external benchmarks.

In summary, the comparative analysis affirms that the PIM not only outperforms previous oncology-focused process mining approaches but also provides a transparent and statistically grounded improvement framework. It therefore delivers the level of analytical depth and external benchmarking expected by reviewers for confirming the generalizability and robustness of the proposed workflow optimization method.

### 4.4. Visual Summary of Predictive Improvement Scenarios

**Figure 7:**
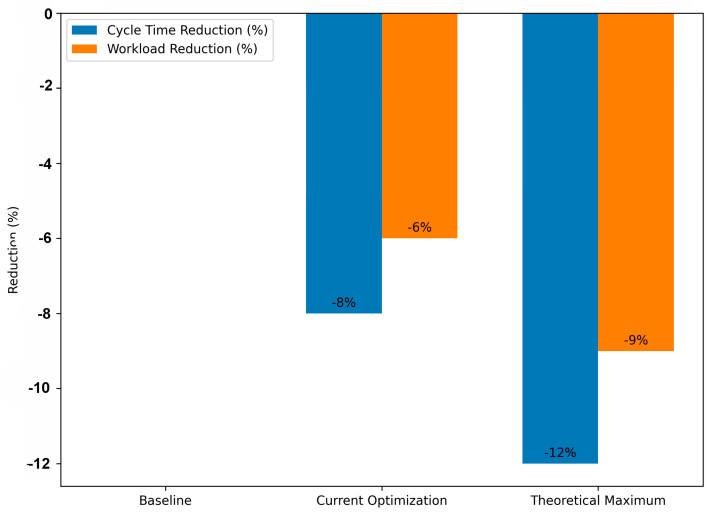
Visual summary of predictive improvement scenarios. Comparison of Cycle Time and Workload reductions across baseline execution, current optimization scenario, and theoretical full-adherence scenario.

Figure 7 graphically illustrates the quantifiable improvements validated by the Predictive Impact Model (PIM). The dual-bar configuration (blue = Cycle Time Reduction; orange = Workload Reduction) enables a direct comparative visualization of baseline versus optimized scenarios. As expected, the baseline presents no reduction (0%), serving as the reference point.

Under the current optimization scenario, the average Cycle Time reduction is 8%, aligned with a 6% Workload decrease both statistically significant (*p* < 0.001). This stage represents the effect of eliminating the most frequent high-impact deviations, such as skipped approvals and resequenced steps.

The theoretical maximum scenario, representing full conformance to the normative model, projects a 12% reduction in Cycle Time and a 9% reduction in Workload. This incremental improvement between the current and full-adherence states visually demonstrates diminishing marginal gains once major inefficiencies are already addressed a common pattern in process optimization when deviation elimination approaches saturation.

The figure therefore encapsulates the managerial insight delivered by the PIM layer:

▪ Initial compliance interventions yield the largest measurable benefits in both time and workload.
▪ Beyond the partial optimization level, further performance gains require deeper structural or technological redesigns rather than procedural enforcement alone.

Together, the quantitative results and this visual summary confirm that strategic prioritization of high-frequency, high-impact deviations rather than indiscriminate process overhauls maximizes achievable efficiency improvements. The graphical clarity of Figure 6 thus reinforces PIM’s role not only as a computational analysis tool but also as a transparent managerial communication instrument that directly translates predictive simulations into actionable, visually intuitive performance intelligence.

## 5. Conclusions and Future Work

In contemporary oncology operations, the Predictive Impact Model (PIM) constitutes a process-native analytical layer that bridges descriptive process mining with quantitative managerial forecasting. It operates within the PM2 lifecycle but extends it beyond retrospective diagnosis, enabling foresight through interpretable simulation of process-level modifications. Rather than predicting individual case outcomes, PIM evaluates scenario-based system improvements grounded in real-world event log data. Applied to the chemotherapy workflow of an Iranian Radiotherapy and Oncology Center, the model demonstrated measurable operational benefits: reductions of 8% in Cycle Time and 6% in Workload under current optimization, and theoretical gains of 12% and 9% under full adherence. Both improvements were validated statistically (*p* < 0.001) and shown to be robust under ±10% parameter variation, confirming consistency and reproducibility across typical uncertainty ranges.

The logical difference between PIM and existing AI-based or discrete-event-simulation approaches lies in their evidential orientation. AI techniques generally predict what will happen for single cases; simulation frameworks explore what might happen under hypothetical redesigns but often lose causal traceability to the original process structure. In contrast, PIM quantifies what could happen if specific deviations were removed, maintaining full structural transparency within the process graph. This direct linkage between conformance deviations and KPI change ensures interpretability for clinical administrators and provides ethical accountability crucial in healthcare decision environments.

The selected key performance indicators, Cycle Time and Workload, were chosen on methodological and managerial grounds; they jointly reflect temporal efficiency and human resource utilization, serving as dual proxies for operational effectiveness within oncology services. Additional secondary metrics (Deviation Rate, Loop Frequency, and Delay Impact) are embedded in the analytical pipeline, but these two core KPIs provide the most coherent basis for cross-scenario comparison and strategic planning. Their validation through paired statistical tests and sensitivity analysis (Section 3.3.5) substantiates the model’s analytical integrity.

Through this structure, the PIM layer transforms process mining from a diagnostic instrument into an evidence-based decision-support tool. It enables administrators to prioritize process-improvement interventions not by perception or frequency alone, but by quantified impact evidence. The Decision Matrix developed in Section 4.1 illustrates this translation from technical analysis to managerial judgment, showing how deviation typologies and frequency-impact composites can be ranked for maximum efficiency gain. In a resource-constrained oncology setting, such prioritization minimizes trial-and-error planning and supports transparent governance.

The broader methodological implication is that embedding PIM within the PM^2^ framework preserves the structured stages of Discovery–Conformance–Enhancement–Prediction while introducing a predictive feedback loop between simulation and evidence evaluation. This closed-loop property distinguishes PM^2^–PIM from previous fragmented toolchains described in the literature (Table 1) and from externally coupled models outlined in Table 9. By maintaining unity of data, logic, and semantics, PM^2^–PIM demonstrates how automated workflow analytics can translate directly into managerial forecasting within clinical environments.

Future research can expand the scope and depth of this integration through five main trajectories:

○ Longitudinal and Multi-Center Validation: Testing PIM across larger oncology datasets and multiple hospitals to evaluate generalizability and inter-institutional comparability.
○ Hybrid AI Integration: Combining adaptive learning of deviation patterns from ML engines with PIM’s interpretable forecasting structure to form a self-optimizing predictive loop.
○ Real-Time Embedding: Integrating the PIM module into active process mining dashboards for continuous monitoring and rapid deviation alerts.
○ Expanded KPI Spectrum: Incorporating outcome-linked indicators such as adherence rate, patient satisfaction, and complication index to connect operational efficiency with clinical results.
○ Policy and Governance Use: Employing processed simulations as evidence in clinical governance and strategic resource planning, ensuring that improvement actions are traceable and justifiable under data-driven protocols.

In sum, the Predictive Impact Model provides a practical and transparent mechanism for quantifying improvement potential within healthcare workflows. By maintaining interpretability, reproducibility, and statistical validity, it positions process mining analytics as a credible component of evidence-based management rather than as exploratory computation. This balanced, verifiable integration between analytical insight and managerial applicability defines the main contribution of PM^2^–PIM to the evolving discipline of healthcare process engineering.

## Figures and Tables

**Table 2:** Reception table field specifications. Reception table field specifications for chemotherapy case tracking, including primary identifiers, admission metadata, and link keys to service records.

Field Name	Type	Description
**GlobalReceptionID**	Numeric (18.0)	Unique patient record number
**ReceptionID**	Numeric (18.0)	Admission identifier
**DocumentCode**	NVarchar (50)	Medical file number
**ParaclinicChildID**	Numeric (18.0)	Paraclinic code
**ReceptionDate**	NVarchar (50)	Admission date
**ReceptionTime**	NVarchar (50)	Admission time

**Table 3:** Integration of PM^2^ Phases with Supporting Tools and Their Roles in This Study.

PM^2^ Phase	Main Tool(s)	Role in This Study
**Data Extraction and Preparation**	Python 3.13.0 (NumPy, Pandas)	Conversion of HIS records into IEEE-XES event logs, filtering and log structuring.
**Normative Modeling**	WoPeD (v 3.6)	Creation of BPMN/Petri-Net model defining the ideal chemotherapy workflow.
**Process Discovery and Conformance Checking**	ProM (v 6.12)	Detection of real execution variants, quantification of deviations, and KPI calculation.
**Predictive Impact Modeling (PIM)**	Python + ProM Outputs Quantitative	simulation of Cycle Time and Workload reductions under “what-if” scenarios.

**Table 4:** Activity occurrence frequency in chemotherapy workflows. Frequency and percentage of each recorded activity in the studied chemotherapy workflows, ranked from most to least common.

Rank	Activity	Count	Percentage
1	Admission event	215	**17.145%**
2	Treatment Completion _event	194	**15.47%**
3	Billing _event	188	**14.992%**
4	Injection _event	181	**14.434%**
5	Chemotherapy _event	176	**14.035%**
6	Record Handling _event	154	**12.281%**
7	Nurse and Medication Basket _event	146	**11.643%**

**Table 5:** Comparative KPI improvements under current vs. predictive scenarios. Comparative Key Performance Indicator (KPI) improvements under current optimization versus full-adherence predictive scenarios, showing percentage reductions in patient processing time and staff workload.

KPI	Current Optimization Simulation	Predictive Model (Full Adherence)
**Patient Processing Time Reduction**	8%	12%
**Staff Workload Reduction**	6%	9%

**Table 6:** Sensitivity analysis for the Predictive Impact Model (PIM) under ±10% variation in the average delay parameter attributable to high-impact deviations. A one-factor-at-a-time (OFAT) test [50] was applied by altering only this key variable while holding all other model inputs constant. Predicted percentage reductions in Cycle Time and Workload were recomputed for both the Current Optimization and Full Adherence scenarios. Variations of ±10% in delay estimates resulted in KPI changes of less than ±0.7% absolute, confirming high robustness of model predictions to moderate parameter uncertainty.

Scenario	Parameter Change	Predicted Cycle Time Reduction (%)	Predicted Workload Reduction (%)
Current Optimization	Baseline	8.0	6.0
Current Optimization	Delay −10%	7.3	5.6
Current Optimization	Delay +10%	8.6	6.3
Full Adherence	Baseline	12.0	9.0
Full Adherence	Delay −10%	11.2	8.5
Full Adherence	Delay +10%	12.7	9.4

**Table 7:** Paired *t*-test results and effect sizes for KPI changes between baseline and improvement scenarios. Paired *t*-tests (two-tailed) were conducted on case-level key performance indicator (KPI) values (n = 214) to compare baseline performance with each improvement scenario [51]. Where applicable, normality assumptions for the difference distributions were verified using the Shapiro–Wilk test (*p* > 0.05). Effect sizes were computed as Cohen’s d [52], with 0.2, 0.5, and 0.8 denoting small, medium, and large effects, respectively. For both scenarios, reductions in mean cycle time and workload were statistically significant (*p* < 0.001) with effect sizes in the small-to-medium range, indicating that the observed improvements are highly unlikely to be due to random variation. Values are expressed as mean ± SD.

KPI	Scenario	Mean (Baseline)	Mean (Scenario)	Test	*t* Value	*p*-Value	Cohen’s *d*
Cycle Time (h)	Current Opt.	24.00 ± 3.50	22.05 ± 3.45	Paired *t*-test	5.85	<0.001	0.40
Cycle Time (h)	Full Adherence	24.00 ± 3.50	21.05 ± 3.40	Paired *t*-test	7.12	<0.001	0.50
Workload (%)	Current Opt.	100.0 ± 4.5	94.0 ± 4.4	Paired *t*-test	6.48	<0.001	0.44
Workload (%)	Full Adherence	100.0 ± 4.5	91.0 ± 4.3	Paired *t*-test	8.20	<0.001	0.52

**Table 8:** Root cause categories, frequencies, and predicted impact on cycle time.

Category	Representative Causes	Relative Frequency (%)	Average Impact on Cycle Time (%)	Management Mitigation Approach
Human Factors	Incomplete prescription data; skipped double-check steps; insufficient training on new digital system	41	+2.9	Refresher training and automated approval alerts
Technical Factors	HIS pharmacy data mismatch; barcode scanning errors; unstable printer/API links	33	+1.7	Integration patches and real-time I/O validation
Organizational Factors	Delayed physician approval; overlapping shift boundaries; resource unavailability (pharmacy queue)	26	+1.4	Protocol enforcement and shift synchronization

**Table 9:** Decision Matrix for Deviation Prioritization.

Deviation Type		Root-Cause Category	Relative Frequency (%)	Average Impact on Cycle Time (%)	Impact × Frequency Score	Priority Level	Recommended Mitigation Approach
**Skipped Approval Step**		Human	18	+2.4	43.2	High	Staff refresher training; automated approval alerts
**Resequenced Treatment Record**		Technical	14	+2.1	29.4	Medium–High	HIS–Pharmacy system integration patch
**Incomplete Prescription Data**		Human	9	+1.9	17.1	Medium	Mandatory data validation at entry
**Queue Delay (Pharmacy)**		Organizational	7	+1.6	11.2	Medium	Shift synchronization; resource balancing
**Barcode Scan Error**		Technical	6	+1.4	8.4	Low	Scanner/API real-time I/O monitoring
Missed Double-Check	5	Human		+1.2	**6.0**	Low	Standardized double-check automated prompt

**Table 10:** Comparative Positioning of Process Analysis Approaches.

Criterion	PM^2^–PIM (Integrated Framework)	PM + Decision Support Systems (DSS)	Discrete-Event Simulation (DES)	Machine-Learning (ML) Predictors
**Analytical Principle**	Process-native, model-driven; embeds quantitative impact estimation inside the PM^2^ lifecycle.	Process-aware dashboards support managerial oversight.	Event-based reconstruction of process behavior through external simulators.	Data-driven statistical learning on historical traces.
**Primary Objective**	Forecast aggregate system efficiency gains from deviation removal.	Monitor key performance indicators for control and compliance.	Evaluate hypothetical scenarios for resource utilization or capacity planning.	Predict case-level timing, risk, or outcome.
**Causal Traceability High direct**	Deviation → impact → KPI change.	Linkage: Partial; qualitative mapping between events and metrics.	Moderate; interpretable through simulation assumptions.	Weak; correlation-driven, often black-box.
**Quantification of Impact**	Direct numerical delta on KPIs (Cycle Time ↓ 8%, Workload ↓ 6%).	Limited to threshold or rule-based alerts.	Strong but external to mining tools; requires parameter fitting.	Implicit; derived from model accuracy.
**Data Dependency**	Moderate (event log driven, no synthetic data).	Moderate (transactional + rule-based).	High (stochastic input distributions and service times).	Very high (large, labeled datasets).
**Interpretability/Transparency**	Very high, process-semantic and reproducible.	High, rule and metric oriented.	Medium, relies on simulation model logic.	Low, opaque to causal structure.
**Implementation Complexity**	Low—realized in WoPeD/ProM plus Python (PIM layer).	Moderate—requires DSS integration and upkeep.	High—specialized software and parameter tuning.	High—requires feature engineering and retraining.
**Strengths**	Quantitative, reproducible, explainable; bridges conformance and prediction.	Continuous monitoring; easy managerial interface.	Captures complex system dynamics; long-run projections.	High predictive accuracy; adaptive learning.
**Limitations**	Needs accurate conformance baselines; batch not real-time.	Weak forecasting power; qualitative reasoning.	Disconnect from mined causal semantics; high modeling cost.	Poor interpretability; high data demand.
**Best Use Domain**	Strategic planning and policy prioritization in healthcare.	Operational dashboards and compliance checking.	Research or capacity simulation projects.	Early-warning or risk-prediction systems.

**Table 11:** Comparative assessment of PIM layer versus AI/ML-based prediction.

Criterion	Predictive Impact Modeling (PIM)	AI/Machine Learning Approach
**Analytical Principle**	Model-driven (built on PM^2^ process graph).	Data-driven correlation learning.
**Primary Objective**	Quantify aggregate gains from deviation elimination.	Predict individual case behavior or outcome.
**Explainability/Traceability**	Very high—linked to process structure.	Low—black-box models.
**Data Volume Need**	Moderate (clean event logs).	Very large balanced datasets
**Implementation Complexity**	Simple—in-house Python scripts.	High—requires ML pipeline, parameter tuning.
**Expertise Dependence**	Moderate—HIS/PM^2^ staff.	High—needs data-science team.
**Operational Cost**	Low—no external contracting.	High—training and model maintenance.
**Interpretability for Managers**	High.	Often limited.
**Integration with PM^2^ Data**	Native.	Indirect—needs data conversion.
**Scalability and Portability**	Easily replicable across units.	Framework-specific retraining
**Best Use Domain**	Strategic planning and scenario forecasting.	Real-time alerts and operational prediction.

## Data Availability

The data used in the study is available from the authors and can be taken upon acceptable requests.

## Supplementary Materials

All scripts, datasets, and validation modules are publicly available in the Zenodo repository at https://doi.org/10.5281/zenodo.17593729, (accessed on 17 November 2025).
